# Inducing Immunity Where It Matters: Orthotopic HPV Tumor Models and Therapeutic Vaccinations

**DOI:** 10.3389/fimmu.2020.01750

**Published:** 2020-08-14

**Authors:** Samantha Zottnick, Alessa L. Voß, Angelika B. Riemer

**Affiliations:** ^1^Immunotherapy and Immunoprevention, German Cancer Research Center (DKFZ), Heidelberg, Germany; ^2^Molecular Vaccine Design, German Center for Infection Research (DZIF), Partner Site Heidelberg, Heidelberg, Germany; ^3^Faculty of Biosciences, Heidelberg University, Heidelberg, Germany

**Keywords:** HPV, orthotopic tumor models, therapeutic vaccination, tissue-resident T cells, MHC-humanized mice

## Abstract

Anogenital and oropharyngeal cancers caused by human papillomavirus (HPV) infections account for 4.5% of all cancer cases worldwide. So far, only the initial infection with selected high-risk types can be prevented by prophylactic vaccination. Already existing persistent HPV infections, however, can currently only be treated by surgical removal of resulting lesions. Therapeutic HPV vaccination, promoting cell-based anti-HPV immunity, would be ideal to eliminate and protect against HPV-induced lesions and tumors. A multitude of vaccination approaches has been tested to date, many of which led to high amounts of HPV-specific T cells *in vivo*. However, growing evidence suggests that not the induction of systemic but of local immunity is paramount for tackling mucosal infections and tumors. Therefore, recent therapeutic vaccination studies have focused on how to induce tissue-resident T cells in the anogenital and oropharyngeal mucosa. These approaches include direct mucosal vaccinations and influencing the migration of systemic T cells toward the mucosa. The efficacy of these new vaccination approaches is best tested *in vivo* by utilizing orthotopic tumor models, i.e. HPV-positive tumors being located in the animal's mucosa. In line with this, we here review existing HPV tumor models and describe two novel tumorigenic cell lines for the MHC-humanized mouse model A2.DR1. These were used for the establishment of an HPV16 E6/E7-positive vaginal tumor model, suitable for testing therapeutic vaccines containing HLA-A2-restricted HPV16-derived epitopes. The newly developed MHC-humanized orthotopic HPV16-positive tumor model is likely to improve the translatability of *in vivo* findings to the clinical setting.

## Introduction

Per year, around 4.5% of new infection-related cancer cases are caused by infections with the human papillomavirus (HPV) (1). While virtually all cervical cancers are caused by HPV-infections, many other anogenital as well as oropharyngeal cancer cases were found to be linked to persistent HPV infections (2). The high-risk type HPV16 is the most abundant HPV type found in HPV-related cancers (3). HPV16 and the second-most frequent high-risk type HPV18 are responsible for 71% of cervical cancer cases worldwide (2, 4).

The HPV oncoproteins E6 and E7 are expressed in all infected cells and are known as the main cause for the induction and maintenance of the malignant phenotype by disrupting cell cycle control in the host cell (5). By leading to the inactivation or even proteasomal degradation of p53 by E6, as well as by inactivation of the retinoblastoma protein (pRb) through E7 activity, a constant viral DNA synthesis is facilitated (6). This impact on infected cells leads to an abnormal cell division, which can result in the development of HPV-mediated cancer. Although E6 and E7 are present in both low- and high-risk types, the binding capacity and effect on cellular signal transduction pathways of these proteins are much stronger in high-risk types (7).

Since 2006, prophylactic vaccines against several HPV types have been available, which were designed with the virus-like particle (VLP) strategy (8, 9). The viral L1 protein self-assembles into empty capsids, which—when used as vaccines—lead to the production of neutralizing antibodies and thereby result in long-lasting protection against the respective HPV types (10). However, as the induced antibodies prevent infection of the target cell by the virus, these vaccines are only effective prior to virus exposure but have no effect against already established HPV infections (11). Current therapeutic approaches are mainly surgical methods with complete removal of the affected tissue, leading to potential severe damages (12). Immunotherapies may provide a non-invasive treatment of already established HPV infections and may further prevent possible lesions caused by new infections. Therapeutic vaccines aim to induce a specific T cell-mediated immune response against HPV-infected cells by targeting HPV-derived epitopes presented by human leukocyte antigen (HLA) molecules on the cell surface (13). Since E6 and E7 are present on both precancerous and advanced cancer stages, they are the most promising target antigens for eliminating infections with high-risk HPV types (4).

For testing the efficacy of a vaccine, a suitable *in vivo* model is required. Papillomaviruses are species-specific, thus HPV does not infect animal cells (14). Most HPV immunotherapy studies were performed in mice with transplantable TC-1 tumor cells. These cells were generated by transduction of C57BL/6 lung cells with HPV16 E6 and E7 as well as H-ras carrying the activating mutation G12V (15, 16). Other tumor models include the C3 cell line (C57BL/6 mouse embryonic cells expressing the HPV16 genome and activated ras) (17), transgenic mouse strains developing tumors (18) and mouse xenograft models (19). The most widely used TC-1 cells only express murine MHC molecules, thus, this model is not suited for testing therapeutic vaccines based on epitopes restricted by HLA molecules. The same applies for the mouse strain C57BL/6. Additionally, although this model can be used for proof of concept studies with murine MHC-restricted HPV16 epitopes, further limitations need to be considered. The TC-1 cell line carries the murine MHC class I molecule H-2D^b^, which presents a highly immunodominant HPV16 epitope (E7_49−57_) (20). Therefore, high frequencies of specific cytotoxic T cells against E7_49−57_ were observed to be induced upon various vaccination approaches, leading to highly efficient killing of TC-1-derived tumors (16, 21). These limitations have been tried to be circumvented with the generation of TC-1/A2 cells, which carry the chimeric HLA-A2 (AAD) molecule (a combination of the epitope-binding α1 and α2 domains of HLA-A^*^0201 with the α3 domain of H-2D^d^) in addition to the murine MHC molecules; and by the use of E7-based vaccines where the immunodominant murine epitope has been mutated (22). To be able to examine HLA-restricted peptides without interference of any epitopes presented on murine MHC molecules, completely HLA-humanized mice were developed. The mouse strain A2.DR1, which expresses the HHD molecule (epitope-binding α1 and α2 domains of HLA-A^*^0201 with the α3 domain of H-2D^b^, covalently bound to human β_2_m) as well as HLA-DR1, is a humanized mouse model expressing the HLA molecules most frequent among Caucasians. Furthermore, all murine MHC genes have been knocked out or rendered inexpressible (23–26).

Another aspect hampering translatability of *in vivo* results is that most HPV16-positive tumor models in mice mainly rely on subcutaneous (s.c.) tumors, but HPV infections occur at mucosal sites with focus on the female genital mucosa. This body site displays a unique immunity which is under the influence of hormonal changes, needs to protect from sexually transmitted infections but must be tolerant to sperm and to a growing fetus (27). Its cell composition differs from that in the peripheral blood (28), and the mucosa is typically not accessed by systemically induced T cells (29). As HPV infections only occur in mucosal epithelia, this is where vaccination-primed specific T cells have to migrate and enter to execute their functions. Thus, in the following, we review current strategies of inducing local anti-HPV immunity at mucosal sites, as well as the development of orthotopic murine HPV tumor models that will allow to assess the anti-tumor efficacy of these vaccination approaches.

## Inducing Anti-HPV Immunity: Systemic vs. Mucosal

In the search for a potent, therapeutic HPV vaccine, a multitude of different formulations has been tested. These include, among others, vaccinations with viral vectors, peptide-based vaccines (minimal epitopes as well as long peptides), whole-protein based vaccines and nucleic acid-based vaccines utilizing RNA as well as DNA (4). Additionally, adoptive cell transfers are under examination (30–32).

These vaccine formulations have been administered in different locations; s.c., intramuscularly (i.m.), as well as intravenously (i.v.). However, most of the studies failed upon their translation to the clinical situation (33). One of the most promising therapeutic HPV vaccinations tested in humans so far has been VGX-3100, which induced high levels of activated CD8^+^ T cells, as well as high antibody titers against the encoded HPV16 and 18 epitopes (34, 35). One possible reason for the observed poor clinical effect probably was the location of the induced immune response, mostly the CD8^+^ T cell-mediated response, which was only assessed systemically.

Upon infection of keratinocytes with HPV, local innate and adaptive immune cells start to produce interferon-γ (IFN-γ). This in turn leads to the production of various IFN-γ-induced chemokines by keratinocytes, including IP-10 (CXCL10) and MIG (CXCL9) (36). Pulled by these chemoattractants, T cells with the respective chemokine receptor, CXCR3 (37), will migrate into the mucosa, where some start to express the integrin CD103. Memory T cells expressing this marker, along with other homing molecules (e.g., CD69), will be retained on-site and are called tissue-resident memory T cells (T_RM_) (38). T_RM_ cells are a vital defense against subsequent reinfections whereupon a rapid T cell response can be initiated. In contrast, circulatory memory T cells will either not enter the mucosae (central memory T cells) or will pass through them without staying (effector memory T cells) (39).

The goal of a therapeutic vaccine against HPV-induced malignancies is the induction of an HPV-specific T cell response, which additionally results in a long-lasting memory response. However, the mucosal localization of the infection hinders activated, circulatory T cells to enter the affected tissue. Therefore, an effective therapeutic immunization against HPV should preferably induce a local immune response in the mucosa, generating T_RM_, rather than systemic immunity to HPV. With this in mind, several approaches have been tested to induce mucosal immunity.

### Local Vaccination

The induction of immunity against HPV in mucosal epithelia can be achieved by directly vaccinating at the site of viral entry. As HPV-derived tumors occur anogenitally, as well as oropharyngeally, vaccination research is focusing on intravaginal, oral and nasal vaccination protocols, checking for induction of local HPV-specific CD8^+^ T cells.

Studies in Lausanne with attenuated *Salmonella enterica* strains expressing HPV16 L1 (40) and later with HPV16 polypeptide vaccines, which induced regression of s.c. tumors (41), showed that the route of delivery influences the type of the induced immune response. Intranasal vaccinations with either vaccine were able to lead to s.c. tumor regression. However, intranasal vaccination with the polypeptide was only able to protect a quarter of tested mice from genital tumors (40, 42). Intravaginal instillation of the *S. enterica* vaccine induced an inflammatory state in the cervicovaginal tract of immunized mice while the adjuvanted peptide vaccination was able to induce a high amount of HPV16-specific CD8^+^ T cells in the cervicovaginal mucosa (42).

When mice were intravaginally inoculated with HPV pseudoviruses consisting of L1, L2 and a pseudogenome expressing a model antigen, the female genital tract was protected from a subsequent challenge with HPV pseudovirus infectious units (IUs) (43). Furthermore, a boost vaccination increased the measured epitope-specific immune response about 10 times. Remarkably, the vaccination also increased the total amount of CD8^+^ T cells in the cervicovaginal tract, with mock-immunized animals not showing this influx of CD8^+^ T cells. Most of the intravaginal T cells were tested positive for the T_RM_ marker CD103 and the protection from infection proved to be long-lasting (43).

The importance of localized immunization was also seen by the induction of a specific T cell response in the oropharyngeal mucosa, where intranasal but not intramuscular vaccination with a non-replicative B unit of Shiga toxin vector (StxB-E7) managed to protect mice from E7-positive head-and-neck tumors. This was shown in both, prophylactic as well as therapeutic immunization settings. The intranasal vaccinations enhanced the amount of HPV16 E7-specific CD8^+^ T cells in the tumor microenvironment of head-and-neck tumors (44). Not only intranasal but also intra-cheek immunizations led to an accumulation of T cells in the local mucosa but not the respective draining lymph node (45).

### Systemic Vaccination

Rather than directly injecting the vaccines into the local tissue, the induction of a systemic T cell response via “classical” routes like s.c., i.m. or i.v. is another possibility to prime the immune system against HPV. These methods, however, must be complemented by directing the T cell migration to the desired location. Over the years, different immune-modulating substances such as Toll-like receptor (TLR)-agonists or chemoattractants have been used not only in HPV research but for several mucosal diseases. The goal is to utilize or induce the expression of mucosa-associated homing molecules on activated T cells.

Shin and Iwasaki first described the so-called “Prime-Pull” method, which mimics the natural occurring immune response (46). Upon infection of vaginal epithelium, effector CD4^+^ T cells enter the tissue and secrete IFN-γ, which in turn leads to the epithelial production of CXCL9 and CXCL10, which attract T cells expressing the homing receptor CXCR3 (47). If administered manually to the vaginal mucosa, these chemokines redirected adoptively transferred, specific CD8^+^ T cells to the mucosa. Mice immunized in this way were protected from a genital herpes simplex 2 infection, whereas mice that received the transferred T cells but were not administered CXCL9 and CXCL10 survived in only 40% of cases (46). Another way of pulling CD8^+^ T cells toward the mucosa is the local application of aminoglycosides such as neomycin. This method is more potent than the chemokine pull and was able to establish a long-lived population of CD69^+^ CD103^+^ positive T cells (48).

Other studies used CpG-ODN (TLR9 agonist) or polyinosinic:polycytidylic acid (poly(I:C), TLR3 agonist) to influence T cell trafficking. The application of either substance led to an increase of the total amount as well as the amount of E7-specific CD8^+^ T cell numbers in the cervicovaginal mucosa of mice after s.c. vaccination with an HPV16 E7 polypeptide vaccine (49). The TLR7 agonist imiquimod was also shown to increase the amount of specific CD8^+^ T cells in the cercivovaginal tract by stimulating the production of IFN-γ and therefore of the chemokines CXCL9 and CXCL10. The accumulation of activated T cells in the mucosa in turn led to further attraction of more CD8^+^ T cells with varying specificity (50). The effect of imiquimod was also replicated in guinea pigs (51).

Another tested compound, all-trans retinoic acid (ATRA) is known to facilitate T cell trafficking to the gut. It has been shown to increase the expression of mucosal homing molecules on T cells as well as leading to an enrichment of functional, specific T cells in the vagina of mice (52).

Rather than pulling with immunomodulators, the vaginal population of epitope-specific T cells can also be enriched by priming the cells systemically and boosting locally. For example, intranasal vaccination with vectors carrying human immunodeficiency virus (HIV) epitopes, followed by a second, vaginal vaccination led to an enlarged population of HIV-specific CD8^+^ CD103^+^ T cells in the vaginal epithelium in mice (53). Similarly, follow-up studies to the HPV pseudovirus vaccinations mentioned above, with adenovirus vectors encoding HPV16 E6 and E7, showed that an i.m. prime vaccination with a subsequent vaginal boost was more effective in inducing HPV-specific CD8^+^ T cells and their trafficking to the cervicovaginal tract than only vaginal vaccination (54, 55).

## Orthotopic HPV Tumor Models

### Existing Orthotopic HPV Tumor Models

Several orthotopic HPV tumor models have been developed to study the vaccination methods mentioned above. The established transplantable HPV16 tumor model TC-1 (C57BL/6 lung cells expressing E6, E7, and H-ras G12V) (15) has been modified for usage in orthotopic studies. Importantly, the cells needed to be transduced with luciferase, to allow monitoring of tumors at body-internal sites, not accessible for caliper measurements. After the transduction with luciferase, the new TC-1-luc cells (56, 57) were used to establish a tumor model in the vagina of C57BL/6 mice. These tumors were monitored *in vivo* via bioluminescence measurements and have been used to test several different vaccination approaches (42, 49, 50, 58, 59). Apart from approaches targeting the induction of local immunity, vaccines inducing systemic immune responses have also been tested using the TC-1-luc model. Interestingly, Bialkowski et al. showed that an intralymphatic vaccine protected mice harboring s.c. or lung tumors better than animals with tumors in the genital tract. This underlines the need to test therapeutic vaccines in orthotopic models that possess a similar microenvironment to naturally occurring tumors in the affected tissue (58). Regression of genital tract TC-1-luc tumors was also induced by vaccination with an i.v. HPV16 RNA-LPX vaccine (59). The cell line has furthermore been used as a model for oropharyngeal cancers and vaccinations by establishing tumors in the submucosal area of the tongue (44) or the submucosal lining of the cheek (45). The HPV16 E6 and E7-positive mEERL95 cell line can also be used as a model for head and neck squamous cell carcinoma (HNSCC) in C57BL/6 mice. It has so far been used to study disease progression after surgical removal of tumors (60). Another transplantable cell line is the C3H-derived AT-84, which was induced to stably express HPV16 E7 and luciferase (61). Therapeutic vaccination with DNA- or plant-based formulations resulted in slowed oral tumor growth of these AT-84 E7-Luc cells.

Other commonly used mouse models are transgenic mice that are engineered to express the HPV proteins of interest, mainly HPV16 and HPV18 E6 and E7 [reviewed in Santos et al. (18)]. If expressed under the cytokeratin 14 promotor, HPV proteins will only be expressed in basal keratinocytes (62). Transgenic models can be used for both, anogenital and oropharyngeal disease modeling (18). However, mice transgenically altered to express HPV proteins may be tolerant to the proteins, leading to the ineffectiveness of therapeutic vaccinations (63).

Recently, newly generated mouse strains were presented whose transgenic expression of HPV16 E6 and E7, as well as mutant K-ras and/or Pten can be induced by instillation of adenoCre virus to vaginal tissue. The genetic modifications led to the development of mucosal tumors which were amenable to endoscopic monitoring, including serial punch biopsies (64). This mouse model can not only be used to track the development of HPV-positive tumors *in vivo* but could also help in developing therapeutic vaccines in the future.

### Development of a Novel Orthotopic HPV16 Tumor Model in MHC-Humanized Mice

The above mentioned orthotopic tumor models can so far only be used for the examination of vaccinations against murine T cell epitopes. Therefore, the translatability from animal model to the clinical stage is limited. To improve the preclinical modeling of HPV16-induced cancers, we established an orthotopic tumor model in the MHC-humanized A2.DR1 mouse model.

**Figure 1:**
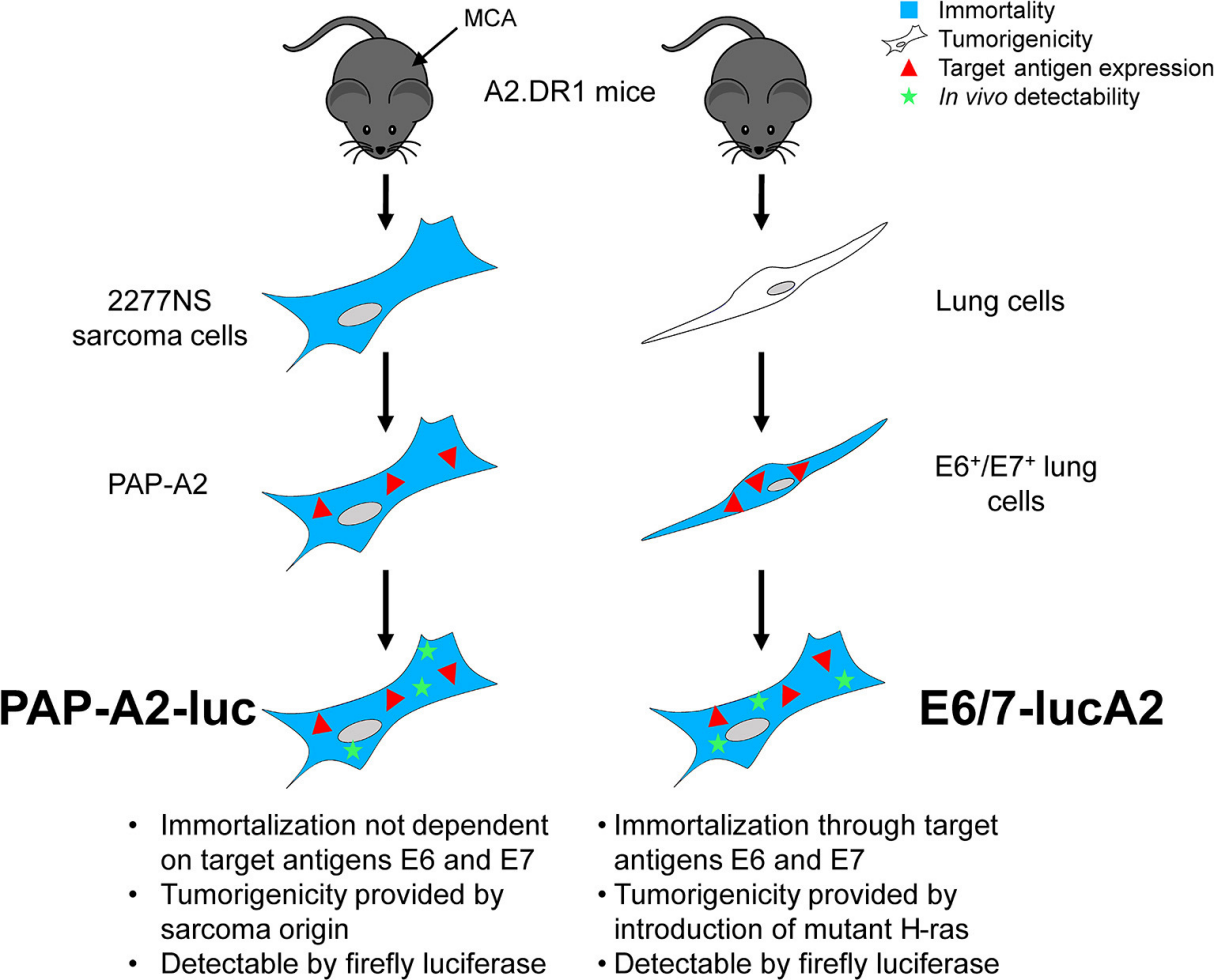
Generation of the two A2.DR1-transplantable HPV16 E6^+^/E7^+^ tumor cell lines PAP-A2-luc and E6/7-lucA2. The A2.DR1-derived sarcoma cell line 2277NS, which was generated by treating mice with methylcholanthrene (MCA) (66), was transduced with a vector carrying the proteins E6 and E7 of HPV16, resulting in the cell line PAP-A2 (65). These cells were transfected with a vector carrying the gene for firefly luciferase, resulting in luminescent PAP-A2-luc cells. The E6/7-lucA2 cell line was generated from isolated murine A2.DR1 lung cells that were transduced with HPV16 E6 and E7, leading to their immortalization and resulting in the expression of the vaccination target antigens. Subsequently, the cells were transfected with mutated H-ras to render them tumorigenic and firefly luciferase to allow tumor monitoring *in vivo*.

The first HPV16 E6^+^/E7^+^ tumor model in A2.DR1 mice (25) was described by our group in 2019 (65) as a target for novel therapeutic HPV vaccination approaches. We generated the E6^+^/E7^+^ cell line PAP-A2 by transduction of the A2.DR1 sarcoma cell line 2277NS with HPV16 E6 and E7. Importantly, E6 and E7 only serve as target antigens in this cell line, which is already immortal and tumorigenic because of its sarcoma origin. In this first study, we worked with s.c. tumors. For the establishment of an orthotopic tumor model we pursued two approaches (Figure 1): one was the further development of the PAP-A2 cell line. It was transfected with the firefly luciferase gene, allowing us to track tumor development *in vivo*. This new cell line was named PAP-A2-luc. The second approach was the establishment of a novel, E6^+^/E7^+^-dependent cell line. To this end, A2.DR1-derived lung cells were transduced with HPV16 E6 and E7. This led to immortalization as well as presence of the desired target antigens. Furthermore, and analogous to TC-1, the cells were transfected with the activated oncoprotein H-ras G12V to render them tumorigenic. Finally, they also received firefly luciferase for *in vivo* detection by luminometers. This cell line was named E6/7-lucA2.

**Figure 2:**
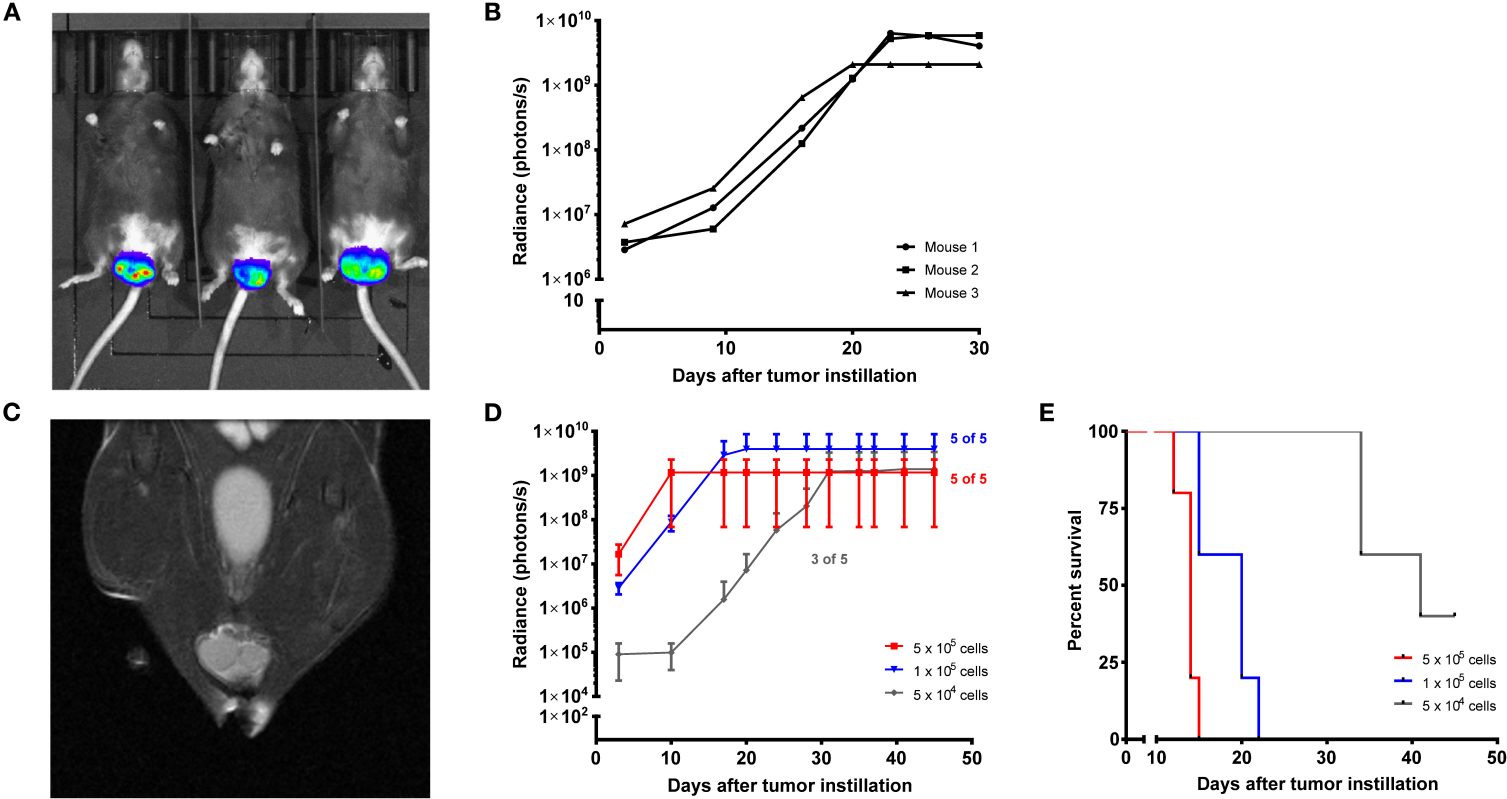
Establishment of E6/7-lucA2 as a novel HPV16 E6^+^/E7^+^ cell line for orthotopic tumor modeling in MHC-humanized A2.DR1 mice. **(A)** Intravaginal tumor growth of E6/7-lucA2 cells. Picture taken 20 days after tumor cell instillation, 7 min after D-luciferin injection i.p. **(B)** Intravaginal tumor growth of mice shown in (A) shown by luminescence over time of instilled E6/7-lucA2 cells. Mice received 1 × 10^6^ cells. **(C)** Magnetic resonance image of a vaginal tumor of a mouse that had received 50,000 E6/7-lucA2 cells intravaginally 22 days prior to imaging, frontal view. **(D)** Orthotopic titration of cell number required for stable tumor formation by intravaginally instilled E6/7-lucA2 cells. Growth shown by luminescence over time. Mean ± SD is shown of 5 mice which received the indicated amounts of cells. **(E)** Cumulative survival curves of groups shown in **(D)**. Survival is defined as the time until mice needed to be sacrificed because reaching one of the pre-specified humane endpoint criteria.

After the expression of the desired proteins HPV16 E6 and E7, H-ras G12V (if applicable) and luciferase was validated by Western blots and luminescence measurements, both cell lines were tested *in vivo* for s.c. tumor growth. Methods are described in Supplementary Table 1. Each cell line was able to form growing s.c. tumors in all tested mice. After re-isolation of the tumor cells, both cell lines were instilled intravaginally into mice that were previously synchronized to a diestrus-like state. PAP-A2-luc tumors grew in 2 of 3 mice (Supplementary Figure 1), while E6/7-lucA2 cells were able to form vaginal tumors in all 3 mice (Figures 2A–C). Because of this and the fact that PAP-A2-luc cells do not depend on the expression of E6 and E7 for survival, we chose to focus on E6/7-lucA2 for further experiments. Therefore, these cells were used to establish the minimal cell number ensuring stable tumor formation and growth. As can be seen in Figures 2D,E, 100,000 cells were sufficient to elicit orthotopic tumor growth, and necessary to achieve this in all animals. This novel orthotopic HPV16 tumor model can now be used to test therapeutic HPV16 vaccination strategies.

## Summary and Conclusion

Therapeutic vaccinations against HPV16-induced lesions and tumors have so far only rarely been effective in clinical trials. One major reason are the shortcomings of preclinical models of HPV-positive tumors, regarding both murine restriction of T cell epitopes and tumor site. As has been shown in recent years, local mucosal immunity, provided by T_RM_ cells, is crucial to fight HPV infection-related malignancies that occur in the anogenital and oropharyngeal mucosa. Therefore, tumor models have to be established at the orthotopic, i.e., naturally occurring, tumor site. Several different orthotopic mouse tumor models have been established, however, they only provide insight into murine immune responses. Our newly developed HPV16 E6^+^/E7^+^ luminescent tumor cell lines, PAP-A2-luc and E6/7-lucA2, for the MHC-humanized mouse model A2.DR1 were shown to be tumorigenic s.c. as well as intravaginally. As the cell line PAP-A2-luc does not rely on the transduced HPV16 proteins for survival and expresses only low levels of these proteins, the E6/E7-dependent cell line E6/7-lucA2 was generated and represents the preferred cell line for future orthotopic tumor experiments in A2.DR1 mice. The advantage of the A2.DR1 mouse strain lies in the exclusive expression of human MHC molecules and can therefore be directly used for vaccination *in vivo*. Regardless of which therapeutic HPV16 vaccinations will be tested in future, our new transplantable tumor cell lines will help to examine their efficacy in a mouse model closely mirroring the clinical setting of HLA-A2-carrying patients. However, a transplantable tumor model cannot depict cancer development and progression. For therapeutic intervention at different stages, other tumor models need to be utilized.

Orthotopic tumor models provide the opportunity to test different vaccination approaches for the induction of tissue-specific immunity. As outlined above, HPV-specific CD8^+^ T cells can either be directly induced in the mucosa by local vaccination or systemically induced T cells can be redirected toward the mucosa by applying appropriate stimuli. In our opinion, the newly developed MHC-humanized orthotopic HPV16-positive tumor model is likely to improve the translatability of *in vivo* findings to the clinical setting.

## Data Availability Statement

All datasets presented in this study are included in the article/supplementary material.

## Ethics Statement

The animal study was reviewed and approved by Regierungspräsidium Karlsruhe.

## Author Contributions

SZ generated both cell lines, and established the E6/7-lucA2 orthotopic tumor model. AV established the PAP-A2-luc tumor model. AR concepted and supervised the experimental study. All authors wrote this manuscript and approved the final version.

## Conflict of Interest

The authors declare that the research was conducted in the absence of any commercial or financial relationships that could be construed as a potential conflict of interest.
