# Supplementary material for: Inducing Immunity Where It Matters: Orthotopic HPV Tumor Models and Therapeutic Vaccinations
Source: Front Immunol. 2020 Aug 14;11:1750. doi: 10.3389/fimmu.2020.01750 (PMC7457000; doi:10.3389/fimmu.2020.01750)
Supplement: Supplementary file 1 [file Image_1.PDF]

Supplementary Figure

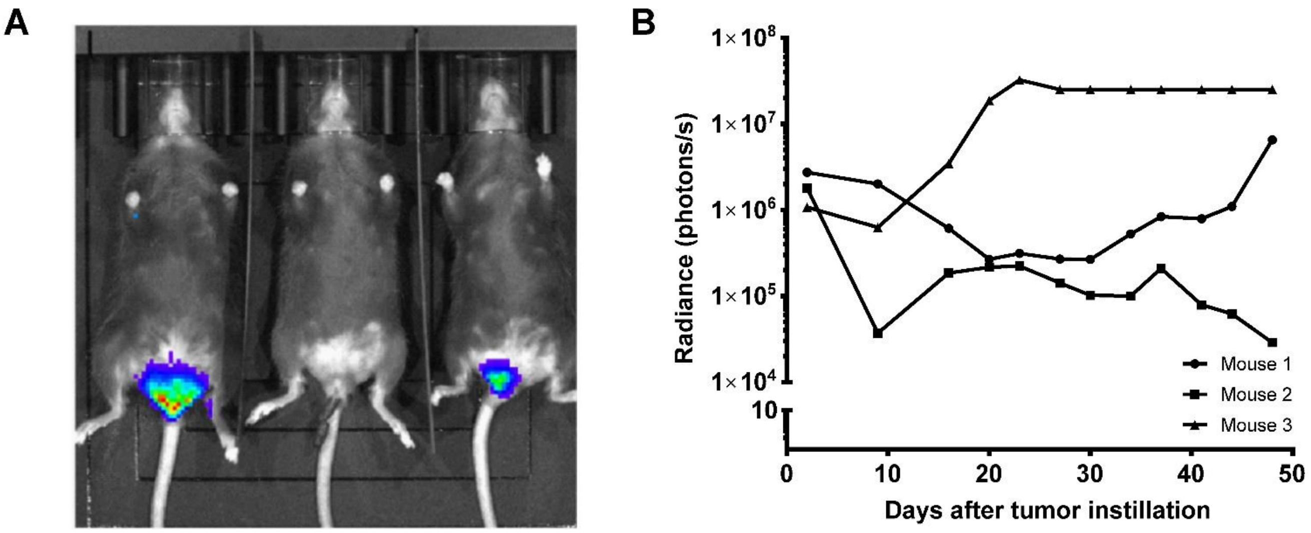

Supplementary Figure 1. Establishment of PAP-A2-luc as a novel HPV16 $E6^{+}/E7^{+}$ cell line for orthotopic tumor modeling in MHC-humanized A2.DR1 mice. (A) Intravaginal tumor growth of PAP-A2-luc cells. Picture taken 9 days after tumor cell instillation, 12 minutes after D-luciferin injection i.p. **(B)** Intravaginal tumor growth of mice shown in (A) shown by luminescence over time of instilled PAP-A2-luc cells. Mice received $1x10^{6}$ cells.
