# Supplementary material for: Inducing Immunity Where It Matters: Orthotopic HPV Tumor Models and Therapeutic Vaccinations
Source: Front Immunol. 2020 Aug 14;11:1750. doi: 10.3389/fimmu.2020.01750 (PMC7457000; doi:10.3389/fimmu.2020.01750)
Supplement: Supplementary file 2 [file Table_1.DOCX]

Supplementary Methods

*Mice*

A2.DR1 mice were provided by the Institut Pasteur (Paris, France) and bred in-house under specific pathogen-free conditions. All national and institutional guidelines were followed and experiments were approved by Regierungspräsidium Karlsruhe. For experiments, female mice aged 6–20 weeks were used.

*Cell lines*

PAP-A2 cells (Kruse et al., 2018) were transfected with a scaffold/matrix attachment region (S/MAR) DNA vector encoding firefly luciferase to generate the PAP-A2-luc cell line. The cells were single-cell sorted, clonally expanded and characterized by Western blot for expression of all desired proteins. One suitable clonal cell line was chosen for further use. Cells were cultured in DMEM supplemented with 10% FBS, 10 mM HEPES buffer, 50 µM β-mercaptoethanol, 4 mM L-glutamine, 1 mM sodium pyruvate, 2 µg/ml puromycin and 2 µg/ml blasticidin.

The cell line E6/7-lucA2 was generated by transducing A2.DR1 lung cells, isolated by digesting lungs with 8 ml of 1 mg/ml collagenase I for 45 minutes, with a lentiviral pWPI vector encoding for tagged versions of full-length HPV16 E6 and E7 and then transfected with an S/MAR DNA vector encoding H-ras G12V and firefly luciferase. The cells were single-cell sorted, clonally expanded and characterized by Western blot for expression of all desired proteins. One suitable clonal cell line was chosen for further use. Cells were cultured in RPMI supplemented with 10 % FBS, 2 mM L-Glutamine, 2 µg/ml puromycin and 1 µg/ml blasticidin.

*Subcutaneous tumor inoculation*

Tumor cells were harvested and washed several times with sterile PBS and 1.5x10^6^ cells were taken up in 50 µl sterile PBS. 50 µl of matrigel were added and the 100 µl resulting solution were injected s.c. in the flank of A2.DR1 mice. Tumors were measured using digital calipers two times a week and tumor volume was calculated using the formula V=4/3*π*((0.5*length [mm])(0.5*width[mm])²).

*Intravaginal tumor instillation*

Mice were synchronized to a diestrus-like state by injecting 100 µl of 1 µg/µl β-estradiol in peanut oil s.c. into the flank 6 days prior to tumor inoculation and 100 µl of 20 mg/ml Depo-Clinovir® (depot medroxyprogesterone acetate) in PBS on the following day. On the day of tumor instillation, 20 µl of 4% nonoxinol-9 were instilled into the vaginal cavity. After 6 hours the vagina was washed several times with PBS and the indicated amounts of tumor cells were instilled in 20 µl PBS/mouse into the vaginal cavity. Mice were sacrificed upon vaginal bleeding or visible signs of distress (e.g. weight loss > 20% of initial weight; ruffled fur, grimacing). Tumor size could not be applied as endpoint as luminescence measurements do not allow for accurate size measurements.

Tumor growth was monitored by luminescence measurements once a week for the first three weeks, afterwards two times a week. Luminescence was examined by injecting intraperitoneally (i.p.) 150 mg/kg body weight of D-luciferin in PBS and subsequent measurement in the IVIS Lumina III Imaging System (PerkinElmer). Analysis was done with the LivingImage® Software (PerkinElmer).

*Magnetic Resonance Imaging (MRI)*

MRI was carried out by the DKFZ small animal imaging core facility using a Bruker BioSpec 3Tesla (Ettlingen, Germany). For imaging, mice were anesthetized with 3.5% sevoflurane in air. For lesion detection, T2 weighted imaging was performed using a T2_TurboRARE sequence: TE = 48 ms, TR = 2000 ms, FOV 30x30 mm, slice thickness 1 mm, averages = 8, Scan Time 6m 24s, echo spacing 12 ms, rare factor 8, slices 9, image size 192 x 192.
